# Role of environmental and occupational factors in fall‐related maxillofacial fractures

**DOI:** 10.1002/cre2.545

**Published:** 2022-04-01

**Authors:** Nigam Sattar, Syeda Rabia Rahat Gillani, Mahwish Erkin, Muslim Khan, Maryam Abbas, Nimrah Khurshid khattak

**Affiliations:** ^1^ Department of Oral Surgery, IIDC Riphah International University Islamabad; ^2^ Department of Oral Surgery Foundation University College of Dentistry Islamabad; ^3^ Department of Oral Surgery Shifa College of Dentistry Islamabad; ^4^ Department of Oral Surgery Khyber College of Dentistry Peshawar; ^5^ AFPGMI Rawalpindi Pakistan; ^6^ Bahria University Medical and Dental College Karachi Pakistan

**Keywords:** dento‐alveolar trauma, environmental factors, falls, maxillofacial fractures, occupational factors

## Abstract

**Objectives:**

The objective of this study was to determine the frequency and patterns of maxillofacial fractures in falls due to environmental and occupational reasons.

**Material and Methods:**

One hundred and nineteen patients were included in this study who presented to the department of Oral and maxillofacial surgery at Khyber College of Dentistry, Peshawar. The duration of study was 3 years from January 1, 2017 to December 31, 2019. Demographic data such as age, gender and data environmental or occupational etiology of falls and pattern of fractures was noted in a customized Performa after detailed history, clinical and radiographic examination. Patients of maxillofacial fractures resulting due to falls with age ranging from 16 to 64 years were included. Those cases of falls that presented with systemic diseases or had chances of pathological fractures were excluded from the study.

**Results:**

Male population was affected more than females (60% vs. 39.4%). The mean age was 32.39 SD ± 16.031. Falls due to environmental factors were more common than occupational factors (81.5% vs 18.5%). Fracture of midface was more common (57.1%) than fracture of mandible (36.7%) in patients of falls. 5.5% had both midface and mandible fractures.

**Conclusion:**

We concluded that Environmental and work‐related facial fractures in falls are common in third and fourth decade of life. Falls due to stumbling, tripping and slipping, falls from height and falls from stairs most commonly result in midface fractures. Mandible fractures are commonly seen in sportsmen and laborers. Preventive strategies shall be adopted to prevent morbidity and mortality associated with such injuries.

## 1 INTRODUCTION

Falls are recognized worldwide as a major threat to the health and lifestyle of older and younger community (Zecevic et al., 2006). It is the leading cause of morbidity and mortality in people above 60 years. Falls result in 2.9 million emergency department visits yearly and 20% of falls cause serious damage such as brain injuries and fractures of maxillofacial region (Ganz et al., 2007; Moncada & Mire, 2017; Tinetti et al., 1994).

Falls can be classified into three categories: Accidental falls (due to external factors, e.g., environmental conditions), predictable physiologic falls (due to intrinsic physiologic factors, such as confusion, attention deficit disorder), unpredictable physiologic falls (due to unexpected intrinsic events, such as a new onset of syncope or major intrinsic event such as stroke, cardiac arrest) (Ganz et al., 2007; Moncada & Mire, 2017; Tinetti et al., 1994).

Environmental causes of falls can be classified as fall due to slippery surfaces for example on snow, fall on ground level due to slipping, tripping, and stumbling, falls from high construction buildings, falls from bed, from trees, from stairs, from rooftops, from mountains falls from hospital beds and wheel chairs (Chen et al., 2016; Roccia et al., 2014; Yamamoto et al., 2010). Workplace related falls can be classified into falls in construction‐workers and falls in sportsmen (Roccia et al., 2013). Socioeconomic factors, low income, illiteracy, occupational hazards and inadequate housing structures with no or minimum prevention are also the risk factors for falls (Li et al., 2013). There is a general consensus that falls probably result from a interaction between internal factors that are specific to every person (e.g., age, gender, general health, behavior, medical illness and activities) and external factors such as person's environment and settings (McVey & Studenski, 1988; Tinetti et al., 1995).

The mechanism by which falls cause facial fractures has evolved over the period of time. The etiology of fall can predict the specific type of injury or fracture. Usually all the fall‐related injuries that include facial fractures, dentoalveolar fractures and soft tissue trauma are affected by the type and severity of falls. Falls from height are usually considered to be high risk for multisystem injury (Velmahos et al., 1997). Head injuries are characteristic of the falls from heights up to 7 and 30 m (McVey & Studenski, 1988). Ground‐level falls (GLF) are considered to be low‐energy type of injury and does not need quick activation of trauma team (Kalula et al., 2016; Zecevic et al., 2006). Most of the falls result in minor soft tissue injuries, but 10%–15% of falls result in fracture and 5% of falls result in more serious soft tissue injury or head trauma (Tinetti et al., 1995). Maxillofacial fractures occur in about 10%–20% of falls. Fractures of the mandible were more frequently noted than middle third of the face. In the mandible, fracture lines were most commonly seen at condyle, especially in simple falls. In the mid face, most frequent bone involvement was seen at the level of zygoma. Nasal bone fractures were also common (Dickinson et al., 2012; Yamamoto et al., 2010) Due to the heavy impact on symphysis or Para symphysis, forces are transmitted indirectly to condyles causing its fracture (Gaddipati et al., 2015). Fractures of the head and neck region due to falls reveal characteristic and predictive features in terms of pattern of injury as well as location and severity of fractures (Yamamoto et al., 2010). Once the pattern of fractures and injury type is identified, management plan is decided by the doctors and hence the hospitalization time is predicted (Nonato, 2011).

Very few studies have focused on predisposing factors like environmental factors and occupational factors that are significant to the risk of falls and the subsequent facial fractures. There is limited published literature regarding the facts and figures of fall‐related maxillofacial injuries in developing countries like Pakistan. Moreover, little is known regarding the risk, safety precautions, training, and experience of fall‐related injuries among expatriate workforce. The objective of this study was to assess the pattern of maxillofacial fractures in falls due to environmental and occupational reasons. This would also help understanding how these etiologies affect the facial bones. It will also in creating safe working environment for sportsmen, laborers as well as for children and elderly population.

## 2 MATERIAL AND METHODS

A prospective cross‐sectional study was conducted at Department of Oral and Maxillofacial Surgery, Khyber College of Dentistry, Peshawar from January 2016 to December 2019. All the cases of maxillofacial fractures; regardless of gender and aged between 16 and 64 as a result of falls due to environmental factors (falls from grounds surface, falls from heights and falls from stairs) and due to occupational factors (construction workers, sportsmen) who reported to Oral and maxillofacial Surgery, Khyber College of Dentistry, Peshawar were included in the study. All the patients were included consecutively. However, falls in Patients due to medical reasons (like syncope, Parkinsonism, epilepsy) were excluded from the study. Approval to carry out the study was sought from the institutional Ethical Review Committee at Khyber College of Dentistry. Informed consent was obtained from all the patients. A detailed history was taken followed by extra oral and intraoral clinical examination and relevant radiographs. This data was collected using a customized Performa which recorded the patient's biographical data as well as the variables such as age, etiology of fall (environmental or occupational), site and pattern of maxillofacial fractures.

The collected data was analyzed using Statistical Package for Social Sciences (SPSS) version 20. Frequencies and percentages were calculated for categorical variables. Mean ± SD was calculated for numerical variables like age. The fractures pattern was stratified among age and etiology of fall. Post‐stratification *χ* ^2^ test was applied keeping *p* value at 0.05 as significant.

## 3 RESULTS

In this study, one hundred and nineteen patients, who had maxillofacial fractures by falls due to environmental and occupational factors were included. Among these, 72 were males (60%) and 47 were females (39.4%). The age range was from 16 to 64 years. The mean age was 32.39 ± 16.031. The common age group was from 16 to 29 years (58%). The patients in middle age group 30–45 were 25.0% and Patients above 45 years were 17%. The details are given in Table 1. The most common etiology of falls was due to environmental factors (Table 2). This was further subdivided into the following:

- Falls due to stumbling, tripping and slipping (22.7%).
- Falls from Stairs (24.7%).
- Falls from Heights more than 3 m (47.4%).
- Falls from heights less than 3 m (5.2). Details are given in Table 3.

**Table 1:** Age distribution of the sample

Age group	Frequency	Percentage
16–29	69	58
30–45	29	25
Above 45	21	17
Total	119	100.0

**Table 2:** Frequency of falls according to etiology

Etiology	Frequency	Percentage
Environmental factors	97	81.5
Occupational factors	22	18.5
Total	119	100

**Table 3:** Frequency of falls due to environmental factors

Environmental factors	Frequency	Percent
Stumbling, slipping and tripping	22	22.7
Fall from stairs	24	24.7
Height > 3 m	46	47.4
Height < 3 m	5	5.2
Total	97	100

The second common cause of falls was due to occupational factors. Among these 18 were construction workers (81.8%) and 4 were sportsmen (18.2%).

Out of 119 patients with Maxillofacial Fractures due to falls, 68 patients had fractures of midface (57.1%) and 44 had mandible fractures (36.7%).7 patients had both midface and mandible fractures (5.5%).

In falls due to environmental factors, falls from height more than 3 m had midface fractures in 73.9% cases, mandible fracture in 10.9% cases and midface and mandible combined fractures in 15.2% cases. Details of fracture pattern stratified by environmental factors and *p* value is given in Table 4. In falls due to occupational Factors, 88.9% of construction workers had mandible fractures, 5.6% had mid face fractures and 5.5% had both midface and mandible fractures. All the sportsmen had mandible fractures (Table 5).

**Table 4:** Frequency of fracture pattern stratified by environmental factors

	Stumbling		Fall by stairs		Height > 3 m		Height < 3 m		Total		
Fracture pattern	*N*	%	*N*	%	*N*	%	*N*	%	*N*	%	*p* Value
Mid face	19	86.4	21	87.5	34	73.9	3	60.6	77	79.4	.039
Mandible	3	13.6	3	12.5	5	10.9	2	40	13	13.4	.02
Both	0	0.0	0	0.0	7	15.2	0	0.0	7	7.2	.018
Total	22	100	24	100	46	100	5	100	97	100	.077

**Table 5:** Frequency of fracture pattern stratified by Occupational Factors

	Construction worker		Sportsmen		Total		
Fracture pattern	*N*	%	*n*	%	*n*	%	*p* Value
Midface	1	5.6	0	0.0	1	4.5	.99
Mandible	16	88.9	4	100	20	91	.818
Midface plus mandible	1	5.5	0	0.0	1	4.5	.98
Total	18	100	4	100	22	100	.783

In the age group 16–29, midface fracture was more common (57.9%) than mandible (31.8%), followed by combined midface and mandible fractures (10.1%). In age group above 45 mandible fracture was equal in number to midface fractures. However, these values were not statistically significant. Details are given in Table 6.

**Table 6:** Frequency of fracture pattern stratified by age groups

	16–29		30–45		Above 45		
Fracture pattern	***N***	**%**	***N***	**%**	***N***	**%**	*p* Value
Mid face	40	57.9	13	54.1	13	50	.027
Mandible	22	31.8	11	45.9	13	50	.0859
Midface and mandible	7	10.1	0	0	0	0.0	.34

## 4 DISCUSSION

Globally, the number of deaths increased by 25% in the last decade and falls claim about 646,000 total deaths. In 1990, falls were the 30th leading cause of death worldwide. By the year of 2013, it became the 28th leading cause of mortality, despite improvement in the healthcare system, better infrastructure, increased literacy rate and more protected environment as compared to past few decades. In the Eastern Mediterranean Region, deaths due to falls have been reported to be 2.8 per 100,000 population which is the highest among World Health Organization regions. Considering morbidities, falls has been reported to be the leading cause of 13.2% of the injury‐related disability, therefore causing huge financial and social problems (Fayyaz et al., 2015).

According to The National Institute of Injury Survey of Pakistan, the annual incidence of injuries due to fall is 8.8 per 1000 population per year. Among them, about 80% of these falls were shown to affect the 20% of adult population. This is a significant public health, economic and social challenge to the country (Bachani et al., 2011). One community‐based survey in Pakistan done in urban, suburban and rural communities showed that fall was one of the most common cause of injuries in older age groups with prevalence of 11 fall injuries per 100 persons in one year (Lasi et al., 2010).

In relation to maxillofacial injuries, fall is third most common of mandible fracture throughout the world while in Pakistan and India it is considered to be the second most common cause (Ashraf et al., 2014).

It is imperative to take the etiology of falls into account. A study conducted in the department of Oral and maxillofacial Surgery, Heidelberg University Hospital from 1997 to 2001 reveals that of 129 patients injured by falling, 34% had fallen from above standing height (stairs) and 26% had fallen in association with acute medical disorders (Lida et al., 2003). In the present study, the most common etiology of falls was due to environmental factors, among which falls from heights more than 3 m was the most common cause, followed by falls from stairs and falls due to tripping slipping and stumbling. These were followed by falls due to occupational causes. This can be justified by that fact that falls due to environmental factors is most common in our country because of lack of proper infrastructure and poor housing conditions. Uneven and unprotected rooftops further add to increase in falls from heights. Little or no attention is paid to develop protective fences in low income housing societies, which frequently result in falls. As no or very little budget is allocated for care of old people by our government, the old homes lack protective environment which result in frequent falls in elderly due to tripping and stumbling (Stenhagen et al., 2013). Study done by Yamamoto et al. report that 26% of injuries occur due to work related accidents (Yamamoto et al., 2010). In the present study, 18% on fractures were seen in work‐place related falls. Etiology of falls due to occupational factors was seen more in laborers and sportsperson.

According to the data collected by NEDSS (National electronic disease survelliance system), among accidental fall patients who presented to emergency department, 69% were between 15 and 44 years of age (Fayyaz et al., 2015). In The current study the most common age group who fell due to environmental and occupational factors was 16–29 years. This can be explained by the fact that people in this age group are socially active. Most of the laborers and sportsmen are in second and third decade of their lives, hence fractures due to falls are seen more commonly in them.

Etiology of falls has a direct impact on the type of injury sustained. The pattern of fracture is influenced by the conditions and mechanism of falls and there is no doubt that falls are a significant cause of oral and maxillofacial fractures. The current study focuses on finding association between type of fall and the facial fracture related with each type of fall. In recent studies done in Italy, falls due to slipping as well as falls from height, the middle third of the face was the most commonly involved region and orbito‐zygomaticomaxillary complex was the most frequent fracture (Roccia et al., 2014). The current study had similar findings. We found midface fractures to be more common in falls due to environmental reasons.

According to Dickinson, “accidental fallers who fall head‐first have higher incidence of neurosurgical lesions (Dickinson et al., 2012). Roccia et al observed comparatively larger percentage of maxillofacial injuries involving the fractures of anterior and posterior tables of the frontal sinus (Roccia et al., 2014). Furthermore, these fractures were seen mostly in those people who fell from heights more than 3 m. Atanasijevic et al observed that the frequency of head injuries is the highest in falls from heights below 7 m (Atanasijevic et al., 2005). This however is in contrast to our findings because most of the patients we received after heights from falls had midface or lower face fractures. This could be explained by the fact the neurological injuries due to falls are usually reported to neurosurgery department so the incidence of skull and upper face injuries due to falls are less commonly reported to maxillofacial units.

Work‐related maxillofacial trauma is rare and often complex. Fabio Roccia and Paolo Boffano carried out a study and found that among 132 patients, 15 patients had fall on the same level and 24 had fall from one level to another. In work‐related injuries, mandible fractures were most common (Goedecke & Thiem, 2019; Roccia et al., 2013) 26This is consistent with The current study as well. The second most common etiological factor of falls in the current study; occupational falls, resulted mostly in mandibular fractures. We concluded that;

- Environmental and occupational factors are most common etiologies of falls.
- Environmental and work‐related facial fractures in falls are common in third and fourth decade of life
- Falls due to stumbling, tripping and slipping, falls from height and falls from stairs most commonly result in midface fractures.
- In sportsmen and laborers, falls mostly cause mandible fractures.
- Maxillofacial fractures resulting from falls, related to environmental and occupational factors are alarming and it's the need of the hour to adopt preventive strategies to prevent morbidity and mortality associated with such injuries.

## AUTHOR CONTRIBUTIONS

*Data collection, manuscript writing, review and editing, critical revision of paper*: Nigam Sattar. *Conception and design, manuscript writing, project administration, approval of final publication*: Muslim Khan. *Data collection and data analysis*: Mahwish Erkin. *Data Analysis and interpretation*: Mariam Abbas.

## CONFLICTS OF INTEREST

The authors declare that there are no conflicts of interest.

## Data Availability

Data is available on request.
